# Acute Effects of Reformer, Cadillac, and Chair Pilates Apparatuses on Cardiac Autonomic Modulation and Flexibility in Sedentary Middle-Aged Women

**DOI:** 10.3390/healthcare14040459

**Published:** 2026-02-11

**Authors:** Ali Kamil Güngör, Hüseyin Topçu, Şenay Şahin, Gökçe Bayram, Monira I. Aldhahi

**Affiliations:** 1 Department of Coaching Education, Faculty of Sport Science, Bursa Uludag University, Bursa 16059, Turkey; alikamilgungor@uludag.edu.tr (A.K.G.); sksahin@uludag.edu.tr (Ş.Ş.); gokcebayram5555@gmail.com (G.B.); 2 Department of Physical Education and Sport Teaching, Faculty of Sport Science, Bursa Uludag University, Bursa 16059, Turkey; huseyintopcu@uludag.edu.tr; 3 Department of Rehabilitation Sciences, College of Health and Rehabilitation Sciences, Princess Nourah bint Abdulrahman University, P.O. Box 84428, Riyadh 11671, Saudi Arabia

**Keywords:** pilates apparatus, cardiac autonomic activity, sit-and-reach, sedentary adults

## Abstract

Background/Objectives: Pilates exercises performed on different apparatuses may elicit distinct acute responses in cardiovascular function and musculoskeletal flexibility, yet comparative data on the immediate effects of reformer (RF), cadillac (CD), and chair (CH) pilates exercises remain unclear. This study aimed to investigate and compare the acute effects of RF, CD, and CH pilates sessions on cardiac autonomic modulation and flexibility in sedentary middle-aged women. Methods: Fifteen participants (mean age: 42.2 ± 1.5 years) completed all three exercise conditions in a randomized crossover design, with sessions separated by at least 72 h. Heart rate variability (HRV) was assessed at pre-exercise, during exercise, and at 10 min intervals up to 40 min post-exercise. Flexibility was measured using standardized sit-and-reach tests at pre-exercise, immediately post-exercise, and 40 min post-exercise. Results: Results revealed a significant condition × time interaction for flexibility (*p* < 0.010, η^2^p = 0.207), with the RF session producing greater immediate improvements in flexibility compared to CD (*p* = 0.030; g = 0.24) and CH sessions (*p* = 0.030; g = 0.24). Notably, flexibility gains from the RF session were maintained at 40 min post-exercise relative to the CH session (*p* = 0.035; g = 0.28). In contrast, no significant interactions between condition × time were observed for HRV parameters (*p* > 0.05). However, the main effect of time was evident across all HRV measures (*p* < 0.05), indicating post-exercise autonomic modulation independent of apparatus type. Conclusions: These findings suggest that while acute cardiovascular responses may not differ substantially between pilates apparatuses, the RF apparatus may be more effective for immediate flexibility enhancement in sedentary middle-aged women. Practitioners and clinicians may consider selecting apparatus based on specific functional goals, such as improving flexibility, when designing pilates-based interventions for this population.

## 1. Introduction

Regular physical activity is associated with substantial health benefits, such as enhanced quality of life, prevention of chronic noncommunicable conditions, and reductions in musculoskeletal and psychological disorders [1]. When appropriately prescribed, exercise enhances physiological tolerance to stress and reduces the risk of cardiovascular disease [1]. Conversely, insufficient physical exercise and a sedentary lifestyle are linked to obesity, postural imbalances, and multiple metabolic or musculoskeletal disorders [2]. These effects are particularly evident in middle-aged women, among whom reduced physical activity accelerates the decline in cardiovascular regulation, neuromuscular coordination, and flexibility, factors that are closely associated with increased cardiovascular disease risk [3].

Among exercise modalities, pilates has gained widespread recognition as a holistic system that integrates physical and mental conditioning [4]. Pilates as a general exercise modality, is based on six core principles, namely breathing, concentration, control, centralization, precision, and rhythm, which together aim to optimize movement efficiency, postural alignment, and mind–body integration [5]. Initially developed to restore functional health, referring to efficient and pain-free execution of daily movements, and body alignment through improved postural control and movement symmetry, pilates emphasizes controlled movements (e.g., slow trunk stabilization exercises such as abdominal plank holds), mindful breathing patterns coordinated with movement, and postural precision aimed at maintaining optimal spinal alignment during exercise. It has been shown to improve several aspects of physical health such as flexibility, muscular endurance, balance, and functional capacity [6,7,8,9], while promoting cardiovascular health in both healthy and clinical populations, including patients with heart failure [10,11]. A meta-analysis reported that pilates improves cardiovascular function in both healthy and clinical populations [10], primarily through its emphasis on respiratory control and neuromuscular coordination. Pilates emphasizes controlled, mindful breathing techniques that have been shown to enhance inspiratory and expiratory muscle strength [12] and improve peripheral circulation efficiency [13]. While mat-based pilates relies mainly on body weight and small equipment, apparatus-based pilates, such as the reformer (RF), cadillac (CD), barrel, and chair (CH), employs adjustable spring resistance to increase external load, movement velocity, and stabilization demands [14]. The RF incorporates a sliding carriage with spring resistance, requiring continuous control of movement acceleration and deceleration across multiple body positions (lying, sitting, or standing), thereby increasing neuromuscular coordination and postural stabilization demands.

In contrast, the CD consists of a fixed four-poster bed equipped with springs and suspended bars, which allows a greater emphasis on supported, assisted, or resisted movements, potentially reducing postural instability while promoting controlled muscle lengthening. The CH apparatus features independent pedals with adjustable springs and a reduced base of support, which may impose greater unilateral loading, balance challenges, and trunk stabilization requirements [15]. From a theoretical perspective, biomechanical differences among pilates apparatuses may elicit distinct autonomic responses by altering stabilization demands, neuromuscular engagement, and mechanical loading, thereby influencing cardiac autonomic modulation and stretch tolerance during and after exercise. However, most prior studies have focused on long-term outcomes or general pilates programs, whereas acute cardiovascular and flexibility responses to specific apparatuses remain poorly understood.

From a physiological standpoint, pilates has been demonstrated to induce transient cardiac autonomic responses, as measured by heart rate variability (HRV), a sensitive marker of parasympathetic and sympathetic activity. HRV is a reliable noninvasive tool for evaluating cardiac autonomic modulation during and after exercise [16] and is defined as the oscillation in the intervals between consecutive R-waves [17]. HRV provides insight into cardiac autonomic adaptability and recovery following exercise, reflecting the capacity for parasympathetic reactivation after physical effort. Autonomic modulation demonstrates the heart’s capacity to adapt to homeostatic challenges rapidly; indeed, autonomic dysfunction is considered a key risk factor for cardiovascular disease and all-cause mortality [18]. Previous studies have demonstrated that pilates may influence HRV by promoting overall autonomic balance rather than isolated vagal activity. For instance, moderate-intensity pilates sessions, typically characterized by an exertion level corresponding to approximately 40–59% of heart rate reserve (HRR) [19], have been shown to reduce R–R intervals and the root mean of the square of the difference in the RR intervals (RMSSD) values in middle-aged women [20], while longer-term equipment-based pilates interventions increased the standard deviation of NN intervals (SDNN) without significant changes in RMSSD in healthy women and men [21,22].

Flexibility is another core pillar of pilates. It plays a crucial role in maintaining musculoskeletal and cardiovascular health [23,24]. Age-related declines in flexibility have been linked to increased arterial stiffness and cardiovascular disease risk, and pilates-based improvements in flexibility may therefore help prevent chronic and cardiovascular diseases in women [25,26]. The controlled, precise movements involved in pilates help individuals develop a greater range of motion in their joints and muscles [24]. Both acute and chronic studies have shown that pilates (whether performed on the mat, with apparatus, or mixed) can enhance flexibility by improving neuromuscular coordination, stretch tolerance, and the viscoelastic properties of muscles and tendons [26,27,28]. Conversely, other investigations report no significant increases in flexibility following a program of pilates compared to RF, mat pilates, and control groups, despite significant within-group flexibility gains observed in RF [29].

Although numerous studies have examined the physiological and functional outcomes of pilates, including blood pressure, cardiac autonomic responses, and flexibility [20,28,29]; most have compared mat-based programs with either RF or mixed apparatus-based protocols [20,29]. To the best of our knowledge, no study has directly compared the acute responses of RF, CD, and CH pilates apparatuses within the same experimental framework. Investigating these distinct modalities is important because each apparatus provides unique mechanical characteristics and resistance profiles that may differentially affect cardiac autonomic regulation and flexibility.

Therefore, the present study aimed to compare the acute effects of RF, CD, and CH pilates exercises on cardiac autonomic modulation and flexibility in sedentary middle-aged women. We hypothesize that RF would be the most beneficial pilates modality for improving both cardiac autonomic modulation and flexibility, compared with CD and CH.

## 2. Materials and Methods

### 2.1. Study Design

**Figure 1:**
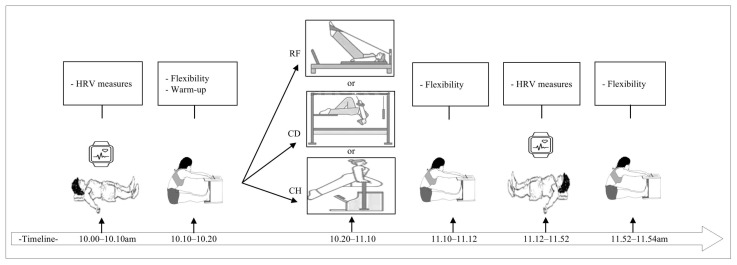
Experimental procedure.

This study employed a randomized, controlled, crossover design. Subjects visited the laboratory on four separate days, with a minimum interval of 72 h between sessions. During the first visit, subjects were familiarized with all experimental procedures and equipment. The second, third, and fourth visits involved three experimental pilates exercise sessions performed on different apparatuses, RF, CD, and CH. A computer-generated randomization sequence was used to assign subjects to three counterbalanced order sequences (*n* = 5 per sequence) at the beginning of the study, using a balanced Latin Square design. This ensured that an equal number of participants started with each pilates apparatus (RF, CD, or CH). In the subsequent sessions, the apparatus order was rotated according to the assigned sequence so that all subjects completed all three exercise protocols in a randomized, crossover design. Through this counterbalanced crossover design, potential order and learning effects were minimized, and each subject served as their own control. HRV was recorded at six time points: pre-exercise, during exercise, and at 10, 20, 30, and 40 min of recovery (rec.-10, rec.-20, rec.-30, rec.-40 min). Flexibility was measured at three time points: pre-exercise, post-exercise, and 40 min after rec. All experimental sessions were conducted under standardized environmental conditions to minimize external influences. The overall experimental procedure is illustrated in Figure 1.

### 2.2. Subjects

Fifteen sedentary middle-aged women aged 40–45 years voluntarily participated in this study (sedentary women were defined as partaking in less than 30 min of moderate-intensity exercise on fewer than three days per week [30]). Subjects were community-dwelling women recruited from a local fitness center (Dream Gym) and were newly enrolled members. A power analysis was conducted using G-Power 3.1.9.7 software (test family: F-test; ANOVA: repeated measures, within-subject factor) with an effect size (f) of 0.30, α = 0.05, and statistical power (1 − β) = 0.80, indicating a minimum sample size of 14. We referenced the study by Holmes et al. [31], which employed a similar research design (with a very large effect size, f = 0.50, and *n* = 10). Accordingly, we selected an effect size of f = 0.30 to avoid overestimation. To account for potential dropouts, 18 subjects were initially recruited; however, three withdrew due to personal reasons, leaving 15 subjects who completed all sessions and were included in the analysis, which provided sufficient power (>80%) to detect the specified effect size [32]. Inclusion criteria were as follows: (i) being female, (ii) aged 40–45 years, (iii) not engaging in regular physical activity, (iv) and having no prior experience with pilates apparatuses. Exclusion criteria included the following: (i) history of metabolic or cardiovascular diseases (e.g., diabetes type 1 or 2, uncontrolled hypertension, arrhythmia); (ii) use of medications known to affect metabolism, heart rate, or blood pressure (e.g., beta-blockers, statins); (iii) current smoking; and (iv) any musculoskeletal limitation preventing exercise participation. All participants provided written informed consent after being thoroughly informed about the study purpose, procedures, potential benefits, and possible risks. The study was conducted in accordance with the Declaration of Helsinki and approved by the Local Clinical Research Ethics Committee (Approval Code: 2025–15/29) and ClinicalTrials.gov Identifier (NCT07308626).

### 2.3. Procedures

All pilates sessions were conducted in the morning, between 10:00 and 11:54 a.m., to control potential circadian variations. Sessions were conducted at the Dream Gym Center (a local gym center) under controlled environmental conditions (temperature: 22–24 °C; relative humidity: 33–45%).

#### 2.3.1. Familiarization Session

All subjects completed a familiarization session prior to the experimental visits. During this session, participants were introduced to all experimental procedures and pilates apparatuses, including the RF, CD, and CH. Anthropometric measurements (body weight, height, body mass index, and body fat percentage) were obtained, and written informed consent was collected. Participants were also familiarized with the HRV recording procedures and the sit-and-reach flexibility test to minimize potential learning effects during experimental sessions.

#### 2.3.2. Experimental Sessions

The first experimental session was performed 72 h after the familiarization session. At the beginning of each session, baseline HRV was recorded during a 10 min pre-exercise resting period, followed immediately by a flexibility assessment using the sit-and-reach test. Subsequently, subjects completed a standardized 5 min warm-up consisting of calisthenic joint mobility exercises (e.g., circular rotations of major joints for approximately 30 s each). Following the warm-up, participants performed a 50 min pilates exercise session using only one apparatus (RF, CD, or CH per visit). HRV was continuously monitored during exercise. Immediately post-exercise, flexibility was reassessed, and subjects were then instructed to remain in the supine position on a mat for 40 min of passive recovery, during which HRV was continuously recorded. A final flexibility assessment was performed at rec.-40 min. The exercises performed on each pilates apparatus, including set and repetition details, are presented in Table 1. Because movements on these pilates apparatuses vary in complexity (e.g., multi-directional vs. simple open–close actions), set durations were not equalized. However, the total exercise time (averaging 50 min) and rest intervals (1 min between exercises) were standardized across all sessions. Exercise intensity was regulated at 40–59% of HRR (considering the calculation to estimate maximum heart rate, 208 − 0.7 × age [33]) to maintain uniform physiological demand across apparatuses. Through continuous real-time monitoring, the instructor adjusted movement tempo to ensure compliance with the target intensity. Resistance loads were standardized to a ‘light-to-moderate’ intensity (typically 1 red or 1 blue spring). This setting was selected to ensure participants could complete the prescribed repetitions with optimal technique, avoiding reaching failure. To minimize the confounding effects of volitional breathing control, participants were instructed to maintain a natural, spontaneous breathing pattern throughout the exercise protocol. Throughout each session, verbal encouragement was provided when necessary to maintain consistent performance. Subjects were instructed to avoid caffeine, alcohol, and strenuous exercise for 24 h, to abstain from food for 3 h, and to avoid fluid intake for 1 h before each session [34]. All sessions were individually delivered and supervised by the same certified pilates instructor, who was experienced in apparatus-based pilates. The research team designed the exercise protocols in accordance with standardized beginner-level (level 1) pilates principles to ensure safety and consistency in participants with no prior pilates experience.

### 2.4. Anthropometric Assessment

Participant height was measured to the nearest 0.1 cm using a stadiometer (Seca 213, Hamburg, Germany). Body composition was assessed using a bioelectrical impedance analysis (BIA) device (Tanita Model BF-350; Tanita Corp., Tokyo, Japan). Prior to the assessment, participants removed their shoes and socks to ensure direct contact with the electrodes and wore light sports clothing to minimize weighing errors. Biological sex, age, and measured height were manually entered into the device. Measurements were recorded using “standard mode”. This setting utilizes the manufacturer’s predictive equations, which are calibrated for the general population. Body mass (kg) was measured via the device’s integrated scale. Upon completion of the bioimpedance analysis, body fat percentage (%), fat mass (kg), and fat-free mass (kg) were recorded. Body mass index (BMI) was calculated as body mass divided by height squared (kg/m^2^).

### 2.5. HRV Assessment

The measurement of HRV through successive R–R intervals was recorded using an HR monitor (Polar V800 with an H10 strap, Polar Electro OY, Kempele, Finland) [35]. HRV data collection was performed at the following time points: (i) pre-exercise: 5–10 min, following a 5 min stabilization period; (ii) exercise: continuously throughout the 50 min session; and (iii) recovery: during 5 min windows at 10, 20, 30, and 40 min post-exercise (corresponding to minutes 5–10, 15–20, 25–30, and 35–40, respectively) [36]. Measurements were performed in the supine position, with subjects remaining quiet, still, and breathing naturally. R–R interval data were subsequently transferred to a computer via the Polar Flow application for analysis. Kubios HRV software (Standard version 3.5.0, Biosignal Analysis and Medical Imaging Group, Department of Physics, University of Kuopio, Kuopio, Finland) was used to analyze HRV parameters. The software automatically performed detrending procedures using smoothness priors [30], removed noise, and applied artifact correction with a very low correction threshold (not exceeding 5% in the current sample). To estimate cardiac autonomic modulation, the following parameters were recorded: Mean-RR represents the average time interval between consecutive R peaks and is inversely related to heart rate; RMSSD predominantly provides information about the parasympathetic system; and SDNN reflects overall HRV and represents both sympathetic and parasympathetic influences [15].

### 2.6. Flexibility (Sit-and-Reach) Assessment

Flexibility was assessed using the sit-and-reach test, which measures flexibility of the lower back and hamstring muscle groups. A standardized sit-and-reach box (35 cm × 45 cm × 32 cm) equipped with a measurement scale was used for this assessment. Participants sat barefoot, legs fully extended and feet together, placing the soles of their feet against the box. From this position, they were instructed to reach forward along the scale with both hands, keeping the knees fully extended, and to hold the furthest reached position for 2 s without altering trunk posture. Each participant performed two trials, and the best score was recorded for analysis [31]. Measurements were obtained at pre-exercise, post-exercise, and 40 min after exercises.

### 2.7. Statistical Analysis

Data analysis was conducted using SPSS version 28.0 software (IBM Corp., Armonk, NY, USA). Descriptive parameters were presented as means and standard deviations. The Shapiro–Wilk test was used to assess the normality of the parameters. A two-way repeated-measures ANOVA (HRV parameters: 3 sessions × 6 trials; flexibility: 3 sessions × 3 trials) was used to evaluate HRV and flexibility parameters. The assumption of sphericity was examined using Mauchly’s test. For factors where the assumption was violated (*p* < 0.05), Greenhouse–Geisser-corrected degrees of freedom were reported. When the assumption was met (*p* > 0.05), sphericity-assumed results were presented. When appropriate, post hoc multiple comparisons were conducted using the Bonferroni adjustment, and the significance level was set at *p* < 0.05. Effect size (ES) was calculated using partial eta squared ($n_{p}^{2}$) (0.01, 0.06, and 0.14 indicating small, medium, and large effects, respectively, ref. [33]) from the repeated-measures ANOVA. Standardized differences were also calculated for pairwise comparisons using Hedges’ g effect sizes with 95% confidence intervals (CIs), which were interpreted based on thresholds outlined by Hopkins et al. [34]: values ≤ 0.19 were considered trivial, ≤0.59 as small, ≤1.19 as moderate, ≤1.99 as large, and ≥2.0 as very large.

## 3. Results

### 3.1. Participant Characteristics

**Figure 2:**
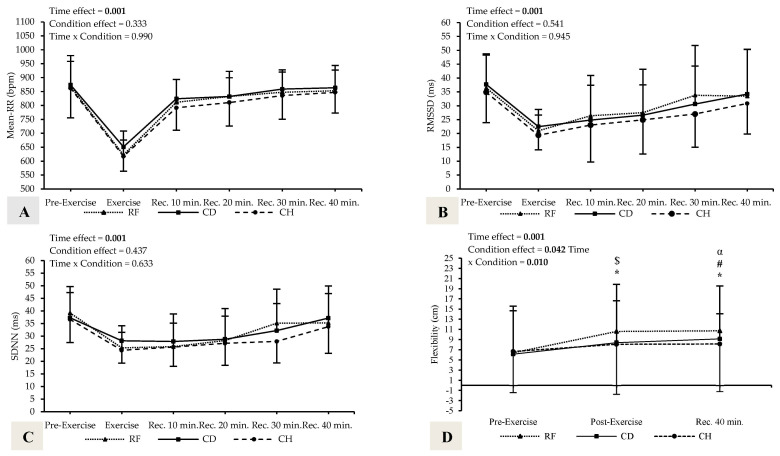
Comparison of HRV and flexibility parameters between sessions and across time. (**A**) Mean-RR responses across RF, CD, and CH sessions; (**B**) RMSSD responses across RF, CD, and CH sessions; (**C**) SDNN responses across RF, CD, and CH sessions; (**D**) Flexibility (sit-and-reach) responses across RF, CD, and CH sessions. * Different from pre-exercise values in RF and CD; ^#^ different from post-exercise values in RF and CD; ^$^ different in post-exercise for RF versus CD and CH; ^α^ different in rec.-40 min for RF versus CH. RMSSD, the root mean of the square of the difference in the RR intervals; SDNN, standard deviation of NN intervals; RF, reformer; CD, cadillac; CH, chair; Pre-ex., pre-exercise; Rec., recovery.

The subjects had a mean age of 42.2 ± 1.5 years, body weight of 68.0 ± 8.2 kg, height of 162.7 ± 6.8 cm, body mass index of 25.7 ± 2.8 kg/m^2^, and body fat percentage of 32.3 ± 3.6. There were no significant differences in baseline characteristics between the experimental sessions (*p* > 0.05). All model effects, along with mean and SD values, are presented in Table 2 and Table 3. Post hoc condition × time plots are presented in Figure 2.

### 3.2. HRV Data

At baseline, no significant differences were observed between the conditions in pre-exercise HRV values (*p* > 0.05). Furthermore, no significant main effect of condition or interaction effect was observed for Mean-RR, RMSSD, and SDNN (*p* > 0.05). Therefore, the data presented below represent the pooled mean values across all three apparatuses to highlight the main effect of time. Post hoc test showed that Mean-RR reduced during exercise (*p* < 0.001; g = 4.15, 95% CI [2.88, 5.42], very large effect) compared to pre-exercise. However, Mean-RR increased rec.-10 min (*p* < 0.001; g = 3.05, 95% CI [−4.10, −2.00], very large effect), rec.-20 min (*p* < 0.001; g = 3.16, 95% CI [−4.24, −2.09], very large effect), rec.-30 min (*p* < 0.001; g = 3.79, 95% CI [−4.98, −2.59], very large effect), and rec.-40 min (*p* < 0.001; g = 3.80, 95% CI [−5.00, −2.60], very large effect) compared to exercise. Post hoc testing indicated that RMSSD decreased during exercise (*p* < 0.001; g = 2.39, 95% CI [1.45, 3.32], large effect), with rec.-10 min (*p* < 0.006; g = 1.37, 95% CI [0.57, 2.17], moderate-to-large effect) and rec.-20 min (*p* < 0.006; g = 1.12, 95% CI [0.35, 1.89], moderate effect) compared to pre-exercise. However, RMSSD increased rec.-30 min (*p* < 0.019; g = 1.14, 95% CI [−1.91, −0.37], moderate effect) and rec.-40 min (*p* < 0.013; g = 1.27, 95% CI [−2.06, −0.49], moderate-to-large effect) compared to exercise. Post hoc test revealed that SDNN reduced during exercise (*p* < 0.001; g = 2.69, 95% CI [1.70, 3.68], large effect), with rec.-10 min (*p* < 0.001; g = 1.93, 95% CI [1.06, 2.80], large effect) and rec.-20 min (*p* < 0.005; g = 1.52, 95% CI [0.71, 2.33], moderate-to-large effect) compared to pre-exercise. However, SDNN increased rec.-40 min (*p* < 0.016; g = 0.88, 95% CI [−1.63, −0.13], moderate effect) compared to exercise.

### 3.3. Flexibility Data

No significant differences were observed between the randomized conditions in pre-exercise flexibility values (all *p* > 0.05). However, unlike the HRV data, a significant condition × time interaction was found for flexibility (*p* < 0.010, η^2^p = 0.207), indicating apparatus-specific responses. Specifically, flexibility increased following the RF both immediately post-exercise (*p* < 0.001; g = 0.44, 95% CI [−1.17, 0.28], small-to-moderate effect) and at rec.-40 min (*p* < 0.001; g = 0.47, 95% CI [−1.20, 0.26], small-to-moderate effect) compared to pre-exercise. Similarly, CD session elicited a significant post-exercise increase (*p* < 0.001; g = 0.26, 95% CI [−0.98, 0.46], small effect) and at rec.-40 min (*p* < 0.001; g = 0.35, 95% CI [−1.08, 0.37], small effect). Moreover, flexibility was significantly higher following RF than both CD (*p* = 0.030; g = 0.24, 95% CI [−0.47, 0.96], small effect) and CH (*p* = 0.021; g = 0.26, 95% CI [−0.46, 0.98], small effect) at post-exercise, and remained greater than CH at rec.-40 min (*p* = 0.035; g = 0.28, 95% CI [−0.44, 1.00], small effect). The main effect of time was observed for flexibility (*p* < 0.001, η^2^p = 0.754). Post hoc test showed that flexibility increased post-exercise (*p* < 0.001; g = 0.29, 95% CI [−1.01, 0.43], small effect) and rec.-40 min (*p* < 0.001; g = 0.34, 95% CI [−1.06, 0.38], small effect) compared to pre-exercise. The main effect of the condition was observed for flexibility (*p* < 0.042, η^2^p = 0.202). Post hoc test showed that flexibility increased in RF (*p* = 0.037; g = 0.18, 95% CI [−0.54, 0.89], trivial-to-small effect) compared to CH.

## 4. Discussion

The present study compared the acute effects of RF, CD, and CH pilates exercises on cardiac autonomic modulation and flexibility in sedentary middle-aged women. The primary finding was that pilates exercise, regardless of the apparatus used, elicited comparable acute cardiac autonomic responses, indicating that the type of apparatus did not meaningfully alter post-exercise autonomic modulation. In contrast, flexibility responses differed across modalities, with RF producing greater immediate improvements than CD and CH apparatus, and these gains were partially maintained during recovery. These findings suggest that while different pilates apparatuses induce similar autonomic stress and recovery patterns, their mechanical characteristics may differentially influence flexibility outcomes.

The findings of the present study are in line with a study by Rocha et al. [16], who investigated the acute effects of pilates exercises on autonomic control in a hypertensive population and showed reductions in R–R interval and rMSSD following a moderate-intensity pilates session. Although Rocha et al. [16], utilized various pilates apparatuses within a single session, the current findings indicate a comparable decrease in autonomic activity, regardless of the specific apparatus used. Notably, autonomic activity returned to the normal range within 40 min in all sessions. These results suggest that pilates exercises performed on the RF, CD, and CH apparatus yield similar impacts on post-exercise autonomic reactivation following an acute session. These findings are also partly supported by Adıgüzel et al. [17], who compared the effects of 10-week equipment-based pilates and diaphragmatic breathing training on HRV and pulmonary function in healthy young women. They observed a significant increase in SDNN, while RMSSD remained unchanged following the pilates intervention, suggesting that pilates may predominantly enhance overall autonomic balance through both sympathetic and parasympathetic adaptations rather than isolated vagal modulation. Consistent results were also reported by Cavina et al. [18], who examined the effects of a 12-week mat-based pilates program in young men. After the intervention, they observed significant improvements in SDNN but no change in RMSSD compared with the control group. Similarly to [17], their results indicate that chronic pilates practice enhances overall autonomic variability without producing marked vagal-specific adaptations. Importantly, both of these studies employed training programs that incorporated a variety of pilates exercises or apparatuses. In contrast, the present study directly compared the acute autonomic responses among different pilates apparatuses under standardized conditions.

It has been proposed that the physiological mechanisms of the pilates method contribute to reduced blood pressure and enhanced cardiorespiratory performance. This effect is primarily attributed to mindful diaphragmatic breathing, which strengthens vagal tone and baroreflex sensitivity. Additionally, the incorporation of smooth and continuous movements sustains metabolic activity and improves endothelial function. Furthermore, the coordinated engagement of multiple muscle groups stimulates nitric oxide release [35]. In the current study, similar responses between sessions may be attributed to the standardized exercise duration, intensity, and the use of basic (level 1) movements, which likely limited the physiological stress imposed by each modality.

In terms of flexibility, all pilates apparatuses produced acute improvements, with the most pronounced gains observed following the RF. This finding suggests that RF may provide a more substantial acute stimulus for enhancing flexibility compared to CD and CH modalities. The present results are consistent with previous research demonstrating the acute and chronic benefits of RF on flexibility. For instance, Pires et al. [22] reported significant improvements in post-exercise flexibility among trained CrossFit practitioners following a single RF-based pilates session, indicating that even highly conditioned individuals can experience acute gains in joint range of motion through the pilates method. Similarly, Bertolla et al. [36] observed immediate post-session increases in flexibility among futsal players after a pilates protocol, highlighting that the method can induce rapid neuromuscular adaptations that enhance stretch tolerance and viscoelastic properties of the muscle–tendon unit. Chronic intervention studies also support the flexibility-enhancing potential of RF. Significant improvements in flexibility among sedentary women were observed after eight weeks of RF [23]. Additionally, significant increases in trunk flexibility and muscular strength in middle-aged women were demonstrated following 12 weeks of mat-based pilates, indicating that both RF and mat modalities promote musculoskeletal adaptations conducive to flexibility enhancement over time [21]. Interestingly, while Yılmaz et al. [24] reported that flexibility increased significantly in the RF group but not in the mat pilates or control groups over an eight-week training period, between-group differences did not reach statistical significance. This may indicate that RF pilates elicits meaningful within-group flexibility gains, though variability in intensity, apparatus use, and participant characteristics that could have influenced between-group outcomes. Although kinematic and muscle activation data were not collected in this study, we hypothesize that the acute superiority of RF over CD and CH apparatuses may be linked to the specific mechanical design of the RF. Unlike the more static nature of the CH and CD, the sliding carriage and spring system of the RF potentially facilitate larger ranges of motion and eccentric loading. It is plausible that this design allows for a combination of active and passive stretching components, contributing to the observed flexibility gains. However, future biomechanical studies are needed to confirm these mechanistic explanations.

Collectively, these findings indicate that although acute cardiac autonomic responses to pilates appear to be largely independent of apparatus type, flexibility responses may vary according to the mechanical and stabilization demands of the modality used. In particular, the RF may provide a more potent acute stimulus for flexibility enhancement, likely due to its capacity to integrate adjustable resistance with controlled, full-range movements. These modality-specific differences may have practical relevance when selecting equipment-based pilates interventions for sedentary middle-aged women.

Several limitations warrant consideration. First, the study population was restricted to sedentary women performing introductory-level exercises, which constrains the generalizability of findings to other demographic groups and exercise intensities. Second, the novice status of all participants introduces the potential for confounding learning effects that could not be entirely eliminated. Third, mechanical workload standardization was compromised by inherent apparatus-dependent variability across modalities. Fourth, the rate of perceived exertion was not systematically quantified, precluding the assessment of subjective effort across conditions. Finally, respiratory patterns were not controlled during data collection to maintain ecological validity; however, this methodological choice limited our ability to isolate the independent effects of breathing frequency on heart rate variability measurements.

## 5. Conclusions

In summary, all pilates apparatuses elicited similar autonomic responses, with transient reductions in HRV followed by full recovery within 40 min. However, flexibility improved across all sessions, with the greatest and most sustained gains observed after the RF. These results suggest that while autonomic effects are independent of apparatus type, the RF may provide an acute stimulus for enhancing flexibility. Therefore, RF-based pilates may be more effective in eliciting acute flexibility improvements in sedentary middle-aged women, though larger and longer-term studies are needed to confirm apparatus-specific physiological responses.

## Figures and Tables

**Table 1:** Exercises were performed with RF, CD, and CH pilates apparatuses.

Reformer				Cadillac			Chair		
No	Exercises	Rep	Set	Exercises	Rep	Set	Exercises	Rep	Set
1	Supine Arm Extension	14	1	Roll Down	14	1	Reverse Swan	14	1
2	Supine Arm Adduction	14	1	The Hundred Beginner	10	1	Teaser	14	1
3	Hundred	10	1	Push Through Bar Abdominal Curl	14	1	Single Leg Teaser	14	1
4	Straight Back	14	1	Teaser	14	1	Mermaid	14	1
5	Side Arm Series/Side Twist	15	1	Trapeze Supine Arm Series	15	1	Side Bend	15	1
6	Rowing Series/T Position	15	1	Pull Down	15	1	Seated Triceps Press	15	1
7	Prone Pulling Straps/T Row	15	1	Chest Expansion	15	1	Prone Scapular Series	15	1
8	Prone Pulling Straps/Arm Extension	15	1	Chest Press	15	1	One Arm Push Ups	15	1
9	Swan	15	1	Swan	15	1	Swan	15	1
10	Shoulder Bridge	15	1	Shoulder Bridge	15	1	Hamstring Curl	15	1
11	Down Circles and Up Circles	15	1	Hip Extension-Hip External Rotation	12	1	Standing Leg Press	15	1
12	Frog	15	1	Supine Leg Spring Series/Leg Circles	15	1	Forward Lunge	15	1
13	Short Box Series/Hamstring Curl	15	1	Frog	15	1	Backward Step Down	12	1
14	Scooter	15	1	Side Lying Leg Springs Series/Leg Adduction	15	1	Side Forward Lunge	15	1

**Table 2:** Comparison of HRV parameters across time and between conditions (*n* = 15).

Variables	Exercise	Pre-Ex.	Exercise	Rec.-10 min.	Rec.-20 min.	Rec.-30 min.	Rec.-40 min.	Time (T) Main Effect	Condition (C) Main Effect	T x C Interactions
		Mean ± SD	Mean ± SD, (Δ%)	Mean ± SD, (Δ%)	Mean ± SD, (Δ%)	Mean ± SD, (Δ%)	Mean ± SD, (Δ%)	$(F_{\left(\mathrm{df}\right)}/n_{p}^{2}$/*P*)	$(F_{\left(\mathrm{df}\right)}/n_{p}^{2}$/*P*)	$(F_{\left(\mathrm{df}\right)}/n_{p}^{2}$/*P*)
Mean-RR	RF	867.66 ± 90.69	622.13 ± 53.54 (−28.3%)	811.53 ± 82.33 (−6.5%)	832.53 ± 89.91 (−4.0%)	847.33 ± 72.63 (−2.3%)	852.86 ± 90.94 (−1.7%)	103.404 (1.74, 24.34) 0.881 0.001 *	1.144 (2, 28) 0.076 0.333	0.250 (2.8, 39) 0.018 0.990
	CD	873.66 ± 105.54	651.20 ± 57.23 (−25.5%)	824.00 ± 69.33 (−5.7%)	832.26 ± 67.35 (−4.7%)	858.73 ± 69.15 (−1.7%)	863.53 ± 63.52 (−1.2%)			
	CH	862.2 ± 106.94	616.93 ± 52.95 (−28.4%)	791.40 ± 80.42 (−8.2%)	810.60 ± 84.15 (−6.0%)	835.33 ± 85.05 (−3.1%)	847.00 ± 74.14 (−1.8%)			
RMSSD (ms)	RF	36.44 ± 12.26	20.98 ± 5.67 (−42.4%)	26.39 ± 14.55 (−27.6%)	27.48 ± 15.69 (−24.6%)	33.79 ± 17.96 (−7.3%)	33.48 ± 16.80 (−8.1%)	14.254 (2.4, 33.21) 0.504 0.001 *	0.628 (2, 28) 0.043 0.541	0.400 (3.5, 48.92) 0.028 0.945
	CD	37.7 ± 10.55	22.44 ± 6.27 (−40.5%)	24.84 ± 12.56 (−34.1%)	26.60 ± 10.93 (−29.4%)	30.62 ± 13.68 (−18.8%)	34.20 ± 16.16 (−9.3%)			
	CH	34.86 ± 10.94	19.36 ± 5.22 (−44.5%)	23.04 ± 13.34 (−33.9%)	24.90 ± 12.28 (−28.6%)	27.01 ± 11.96 (−22.5%)	30.81 ± 11.03 (−11.6%)			
SDNN (ms)	RF	39.2 ± 10.53	25.35 ± 6.17 (−35.3%)	25.87 ± 9.28 (−34.0%)	28.24 ± 12.73 (−28.0%)	35.15 ± 13.55 (−10.3%)	35.28 ± 11.67 (−10.0%)	14.864 (2.33, 32.64) 0.515 0.001 *	0.852 (2, 28) 0.057 0.437	0.796 (3.5, 48.94) 0.054 0.633
	CD	37.28 ± 10.01	28.16 ± 5.98 (−24.5%)	27.90 ± 10.88 (−25.2%)	28.77 ± 9.15 (−22.8%)	32.20 ± 10.77 (−13.6%)	37.16 ± 12.78 (−0.3%)			
	CH	36.64 ± 9.16	24.38 ± 5.08 (−33.5%)	25.66 ± 7.64 (−30.0%)	27.15 ± 8.72 (−25.9%)	27.89 ± 8.54 (−23.9%)	33.78 ± 10.59 (−7.8%)			

* Significant difference (*p* < 0.05). Δ% indicates percentage change relative to the pre-ex. values. RMSSD, the root mean of the square of the difference in the RR intervals; SDNN, standard deviation of NN intervals; RF, reformer; CD, cadillac; CH, chair; Pre-ex., pre-exercise; Rec., recovery.

**Table 3:** Comparison of flexibility across time and between conditions (*n* = 15).

Variables	Exercise	Pre-Exercise	Post-Exercise	Rec.-40 min.	Time (T) Main Effect	Condition (C) Main Effect	T x C Interactions
		Mean ± SD (Δ%)	Mean ± SD (Δ%)	Mean ± SD (Δ%)	$(F_{\left(\mathrm{df}\right)}/n_{p}^{2}$/ *P*)	$(F_{\left(\mathrm{df}\right)}/n_{p}^{2}$/ *P*)	$(F_{\left(\mathrm{df}\right)}/n_{p}^{2}$/*P*
Flexibility (cm)	RF	6.4 ± 9.15	10.6 ± 9.26 (+65.6%)	10.73 ± 8.8 (+67.7%)	42.975 (2, 28) 0.754 0.001 *	3.544 (2, 28) 0.202 0.042 *	3.661 (2.76, 38.6) 0.207 0.010 *
	CD	6.13 ± 8.53	8.4 ± 8.24 (+37.0%)	9.13 ± 4.95 (+48.9%)			
	CH	6.66 ± 8.09	8.06 ± 9.83 (+21.0%)	8.13 ± 9.38 (+22.1%)			

* Significant difference (*p* < 0.05). Δ% indicates the percentage change relative to the pre-exercise values. RF, reformer; CD, cadillac; CH, chair; Rec., recovery.

## Data Availability

The data presented in this study are available upon request from the corresponding author. The data are not publicly available for further publication because of restrictions related to the ongoing work.

## References

[1] Lee I.M., Shiroma E.J., Lobelo F., Puska P., Blair S.N., Katzmarzyk P.T., Alkandari J.R., Andersen L.B., Bauman A.E., Brownson R.C. (2012). Effect of Physical Inactivity on Major Non-Communicable Diseases Worldwide: An Analysis of Burden of Disease and Life Expectancy. Lancet.

[2] Seong D., Kim D.S. (2025). The Effects of Mat Pilates Exercise and Barrel Pilates Exercise on Body Composition and Muscle Activity in Adult Women. J. Bodyw. Mov. Ther..

[3] Ruberti O.M., Sousa A.S., Viana L.R., Pereira Gomes M.F., Medeiros A., Gomes Marcondes M.C.C., de Borges L.F., Crestani C.C., Mostarda C., da Cunha Moraes T.F. (2021). Aerobic Training Prevents Cardiometabolic Changes Triggered by Myocardial Infarction in Ovariectomized Rats. J. Cell. Physiol..

[4] Pereira M.J., Mendes R., Mendes R.S., Martins F., Gomes R., Gama J., Dias G., Castro M.A. (2022). Benefits of Pilates in the Elderly Population: A Systematic Review and Meta-Analysis. Eur. J. Investig. Health Psychol. Educ..

[5] Bird M.L., Hill K.D., Fell J.W. (2012). A Randomized Controlled Study Investigating Static and Dynamic Balance in Older Adults after Training with Pilates. Arch. Phys. Med. Rehabil..

[6] De Oliveira L.C., De Oliveira R.G., De Almeida Pires-Oliveira D.A. (2015). Effects of Pilates on Muscle Strength, Postural Balance and Quality of Life of Older Adults: A Randomized, Controlled, Clinical Trial. J. Phys. Ther. Sci..

[7] Markovic G., Sarabon N., Greblo Z., Krizanic V. (2015). Effects of Feedback-Based Balance and Core Resistance Training vs. Pilates Training on Balance and Muscle Function in Older Women: A Randomized-Controlled Trial. Arch. Gerontol. Geriatr..

[8] Rayes A.B.R., De Lira C.A.B., Viana R.B., Benedito-Silva A.A., Vancini R.L., Mascarin N., Andrade M.S. (2019). The Effects of Pilates vs. Aerobic Training on Cardiorespiratory Fitness, Isokinetic Muscular Strength, Body Composition, and Functional Tasks Outcomes for Individuals Who Are Overweight/Obese: A Clinical Trial. PeerJ.

[9] Fernández-Rodríguez R., Álvarez-Bueno C., Ferri-Morales A., Torres-Costoso A.I., Cavero-Redondo I., Martínez-Vizcaíno V. (2019). Pilates Method Improves Cardiorespiratory Fitness: A Systematic Review and Meta-Analysis. J. Clin. Med..

[10] Guimarães G.V., Carvalho V.O., Bocchi E.A., d’Avila V.M. (2012). Pilates in Heart Failure Patients: A Randomized Controlled Pilot Trial. Cardiovasc. Ther..

[11] Bağlan Yentür S., Saraç D.C., Sari F., Tore G., Bilici Salman R., Akif Öztürk M., Oskay D. (2024). The Effects of Pilates Training on Respiratory Muscle Strength in Patients with Ankylosing Spondylitis. Physiother. Theory Pract..

[12] Tinoco-Fernández M., Jiménez-Martín M., Sánchez-Caravaca M.A., Fernández-Pérez A.M., Ramírez-Rodrigo J., Villaverde-Gutiérrez C. (2016). The Pilates Method and Cardiorespiratory Adaptation to Training. Res. Sports Med..

[13] Lee K. (2021). The Relationship of Trunk Muscle Activation and Core Stability: A Biomechanical Analysis of Pilates-Based Stabilization Exercise. Int. J. Environ. Res. Public Health.

[14] Stanley J., Peake J.M., Buchheit M. (2013). Cardiac Parasympathetic Reactivation Following Exercise: Implications for Training Prescription. Sports Med..

[15] Malik M. (1996). Heart Rate Variability. Ann. Noninvasive Electrocardiol..

[16] Rocha J., Cunha F.A., Cordeiro R., Monteiro W., Pescatello L.S., Farinatti P. (2020). Acute Effect of a Single Session of Pilates on Blood Pressure and Cardiac Autonomic Control in Middle-Aged Adults with Hypertension. J. Strength Cond. Res..

[17] Adıgüzel S., Aras D., Gülü M., Aldhahi M.I., Alqahtani A.S., AL-Mhanna S.B. (2023). Comparative Effectiveness of 10-Week Equipment-Based Pilates and Diaphragmatic Breathing Exercise on Heart Rate Variability and Pulmonary Function in Young Adult Healthy Women with Normal BMI—A Quasi-Experimental Study. BMC Sports Sci. Med. Rehabil..

[18] Cavina A.P.S., Silva N.M., Biral T.M., Lemos L.K., Junior E.P., Pastre C.M., Vanderlei L.C.M., Vanderlei F.M. (2021). Effects of 12-Week Pilates Training Program on Cardiac Autonomic Modulation: A Randomized Controlled Clinical Trial. J. Comp. Eff. Res..

[19] Das T., Bandyopadhyay N. (2023). Pilates Exercises, Types, and Its Importance: An Overview. World Congress on Multi Disciplinary Cohesion for Positive Health and Well Being.

[20] Kloubec J. (2011). Pilates: How Does It Work and Who Needs It?. Muscles Ligaments Tendons J..

[21] Kao Y.H., Liou T.H., Huang Y.C., Tsai Y.W., Wang K.M. (2015). Effects of a 12-Week Pilates Course on Lower Limb Muscle Strength and Trunk Flexibility in Women Living in the Community. Health Care Women Int..

[22] Pires K.A., Rocha D.S., Gotti Alves R.R., Silva O.O., Bertolini G.R.F., Bertoncello D. (2024). Acute Effects of a Pilates Method Session on Flexibility and Performance in Practitioners of an Extreme Conditioning Program: A Preliminary Study. J. Bodyw. Mov. Ther..

[23] Suna G., Işildak K. (2020). Investigation of the Effect of 8-Week Reformer Pilates Exercise on Flexibility, Heart Rate and Glucose Levels in Sedentary Women. Asian J. Educ. Train..

[24] Yılmaz O., Kaplan T., Batalik L. (2025). Randomised Controlled Study on the Effects of Pilates Exercises in Soccer: Comparing Mat and Reformer Methods on Physical and Technical Performance. PLoS ONE.

[25] Holmes C.J., MacDonald H.V., Esco M.R., Fedewa M.V., Wind S.A., Winchester L.J. (2022). Comparison of Heart Rate Variability Responses to Varying Resistance Exercise Volume-Loads. Res. Q. Exerc. Sport.

[26] Beck T.W. (2013). The Importance of a Priori Sample Size Estimation in Strength and Conditioning Research. J. Strength Cond. Res..

[27] Christiani M., Grosicki G.J., Flatt A.A. (2021). Cardiac-Autonomic and Hemodynamic Responses to a Hypertonic, Sugar-Sweetened Sports Beverage in Physically Active Men. Appl. Physiol. Nutr. Metab..

[28] Giles D., Draper N., Neil W. (2016). Validity of the Polar V800 Heart Rate Monitor to Measure RR Intervals at Rest. Eur. J. Appl. Physiol..

[29] Esco M.R., Flatt A.A. (2014). Ultra-Short-Term Heart Rate Variability Indexes at Rest and Post-Exercise in Athletes: Evaluating the Agreement with Accepted Recommendations. J. Sports Sci. Med..

[30] Tarvainen M.P., Ranta-aho P.O., Karjalainen P.A. (2002). An Advanced Detrending Method with Application to HRV Analysis. IEEE Trans. Biomed. Eng..

[31] Rikli R., Jone J. (2013). Senior Fitness Test Manual.

[32] Hopkins W.G., Marshall S.W., Batterham A.M., Hanin J. (2009). Progressive Statistics for Studies in Sports Medicine and Exercise Science. Med. Sci. Sports Exerc..

[33] Latey P. (2001). The Pilates Method: History and Philosophy. J. Bodyw. Mov. Ther..

[34] Marasingha-Arachchige S.U., Rubio-Arias J., Alcaraz P.E., Chung L.H. (2022). Factors That Affect Heart Rate Variability Following Acute Resistance Exercise: A Systematic Review and Meta-Analysis. J. Sport Health Sci..

[35] Barbosa R.F., Gomes M.F.P., Ruider D.T., Rodrigues B., Barbosa A.C.B., Dourado V.Z., Medeiros A. (2025). The Pilates Method Reduces Blood Pressure and Increases Respiratory Strength and Cardiorespiratory Capacity in Medicated Hypertensive Women. Eur. J. Appl. Physiol..

[36] Bertolla F., Baroni B.M., Leal Junior E.C.P., Oltramari J. (2007). Effects of a Training Program Using the Pilates Method in Flexibility of Sub-20 Indoor Soccer Athletes. Rev. Bras. Med. Esporte.

